# Supplementary material for: N6-methyladenosine modification governs liver glycogenesis by stabilizing the glycogen synthase 2 mRNA
Source: Nat Commun. 2022 Nov 17;13:7038. doi: 10.1038/s41467-022-34808-2 (PMC9671881; doi:10.1038/s41467-022-34808-2)
Supplement: Supplementary file 1 — Supplementary Information [file 41467_2022_34808_MOESM1_ESM.pdf]

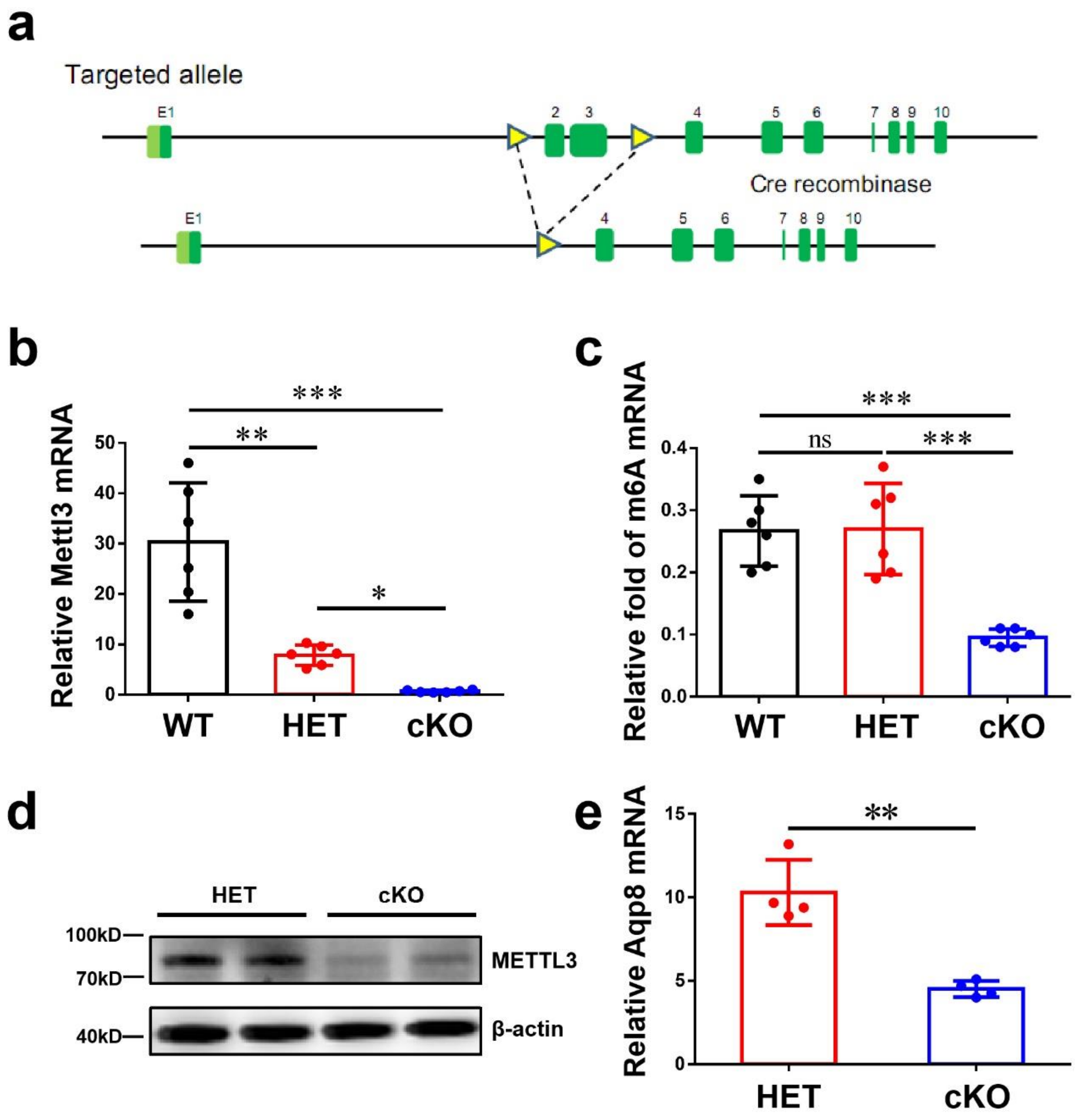

supplementary Figure 1. Generation of hepatocyte-specific *Mettl3* knockout mice. (a) Schematic diagram of strategy to construct flox mouse. (b) qRT-PCR assay of *Mettl3* mRNA level (β-actin as reference) in wildtype (WT), heterozygous (HET) and conditional Knockout (cKO). n=6 animals. Data are presented as mean values +/- SEM. One-way ANOVA was performed to determine a difference between each column. *, $p < 0.05$; **, $p < 0.01$; ***, $p < 0.001$. (c) The levels of m6A mRNA were analyzed in livers of different genotypes. n=6 animals. Data are presented as mean values +/- SEM. One-way ANOVA was performed to determine a difference between each column. ns, not significant; ***, $p < 0.001$. (d) Western blotting assay of *Mettl3* protein level in HET and cKO mouse livers. This experiment was repeated independently with similar results at least 3 times. (e) qRT-PCR assay of *Aqp8* mRNA level (β-actin as reference) in HET and cKO mouse livers. n=4 animals. Data are presented as mean values +/- SEM. Two-sided Student's t test was performed to determine a difference among groups, **, $p = 0.001$. Source data are provided as a Source Data file.

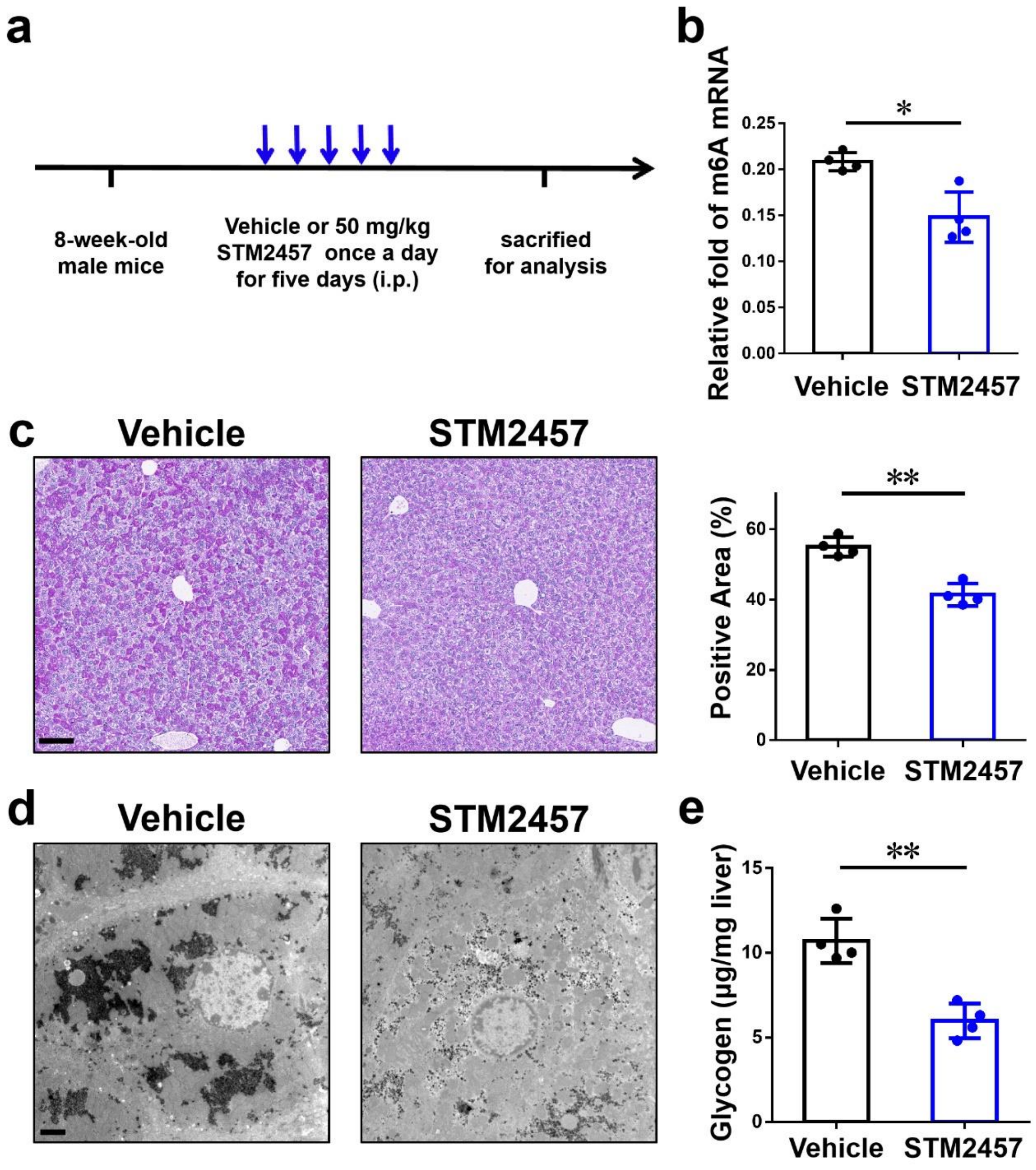

supplementary Figure 2. Pharmacological inhibition of METTL3 simulates lack of glycogen in liver. (a) Schematic diagram of strategy to treat mice with vehicle or STM2457. (b) The levels of m6A mRNA were analyzed in livers of different groups. n=4 animals. Data are presented as mean values +/- SEM. Two-sided Student's t test was performed to determine a difference among groups. *, p = 0.03. (c) PAS staining of livers in 8-week-old mice treated by vehicle or STM2457. The percentage of positive area is measured by Image J and shown on the right. Bar, 100μm. n=4 animals. Data are presented as mean values +/- SEM. Two-sided Student's t test was performed to determine a difference among groups. **, p = 0.007. (d) Transmission electron microscope pictures of livers in 8-week-old mouse treated by vehicle or STM2457. Bar, 2μm. This experiment was repeated independently with similar results at least 3 times. (e) Hepatic glycogen content of mice treated by vehicle or STM2457. n=4 animals. Data are presented as mean values +/- SEM. Two-sided Student's t test was performed to determine a difference among groups. **, p =

0.002. Source data are provided as a Source Data file.

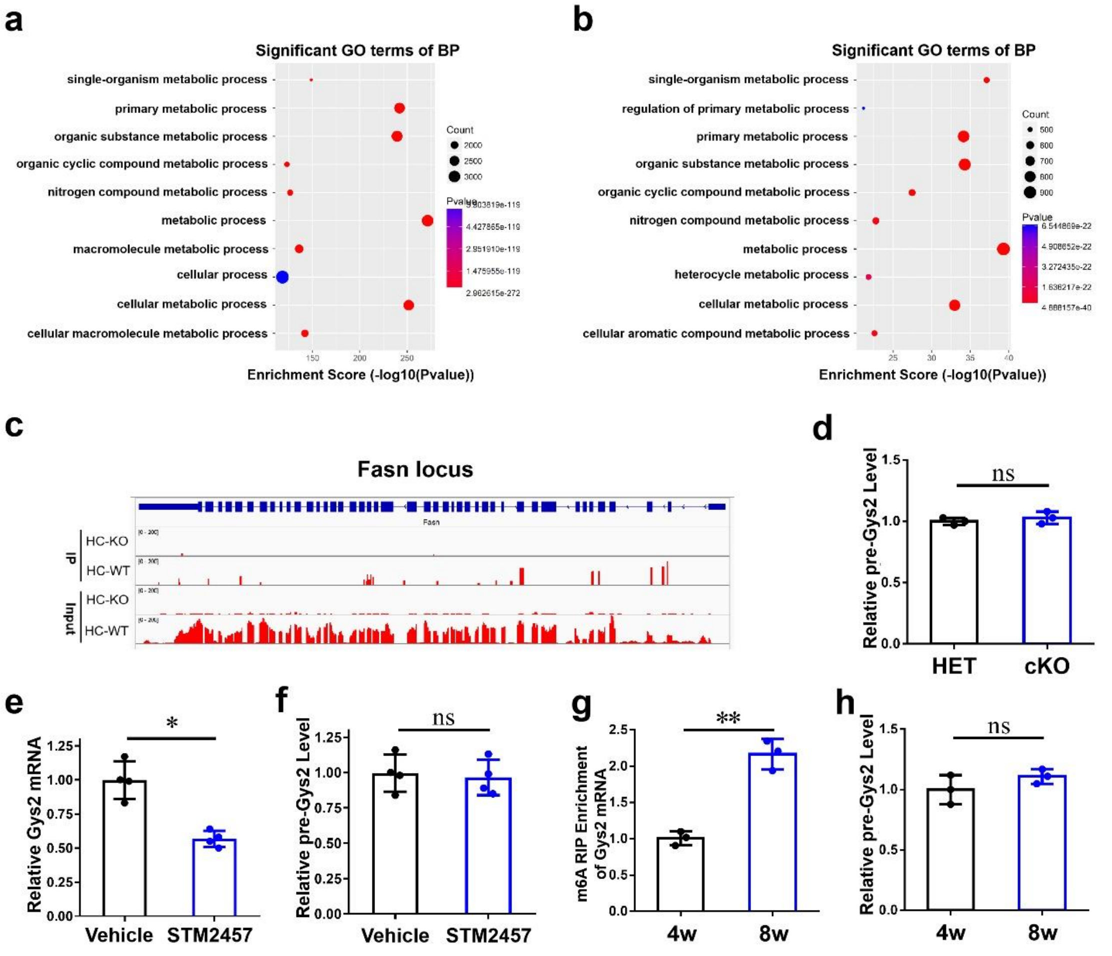

supplementary Figure 3. related to Figure 3. Identification of *Gys2* mRNA by global N6-methyladenosine modification analysis as a key substrate of METTL3 in mouse liver. (a-b) Significantly enriched (p-value ≤ 0.01, Benjamini–Hochberg multiple testing correction) GO terms of genes with m6A peak loss (a) and down-regulated expression (b) in *Mettl3*-cKO liver. Kolmogorov-Smirnov test was performed to determine significance. (c) m6A MeRIP-Seq revealed the location of specific m6A peak in *Fasn* locus in hepatocytes of wildtype or Mettl3-cKO mice. (d) qRT-PCR of pre-Gys2 (nascent) mRNA levels (18s as reference) in 8-week-old mouse livers with indicated Mettl3 genotypes. n=3 animals. Data are presented as mean values +/- SEM. Two-sided Student's t test was performed to determine a difference among groups. ns, not significant. (e) qRT-PCR of mature Gys2 mRNA levels (β-actin as reference) in 8-week-old mouse livers treated with vehicle or STM2457. n=4 animals. Data are presented as mean values +/- SEM. Two-sided Student's t test was performed to determine a difference among groups. *, $p = 0.02$. (f) qRT-PCR of pre-Gys2 (nascent) mRNA levels (β-actin as reference) in 8-week-old mouse livers treated with vehicle or STM2457. n=4 animals. Data are presented as mean values +/- SEM. Two-sided Student's t test was performed to determine a difference among groups. ns, not significant. (g) m6A enrichment of *Gys2* mRNA in hepatocytes of 4-week-old or 8-week-old wildtype mice by m6A-RIP-qPCR. n=3 animals. Data are presented as mean values +/- SEM. Two-sided Student's t test was performed to determine a difference among groups.

**, p = 0.009. (h) qRT-PCR of pre-Gys2 (nascent) mRNA levels (18s as reference) in wildtype mouse livers with indicated ages. n=3 animals. Data are presented as mean values +/- SEM. Two-sided Student's t test was performed to determine a difference among groups. ns, not significant. Source data are provided as a Source Data file.

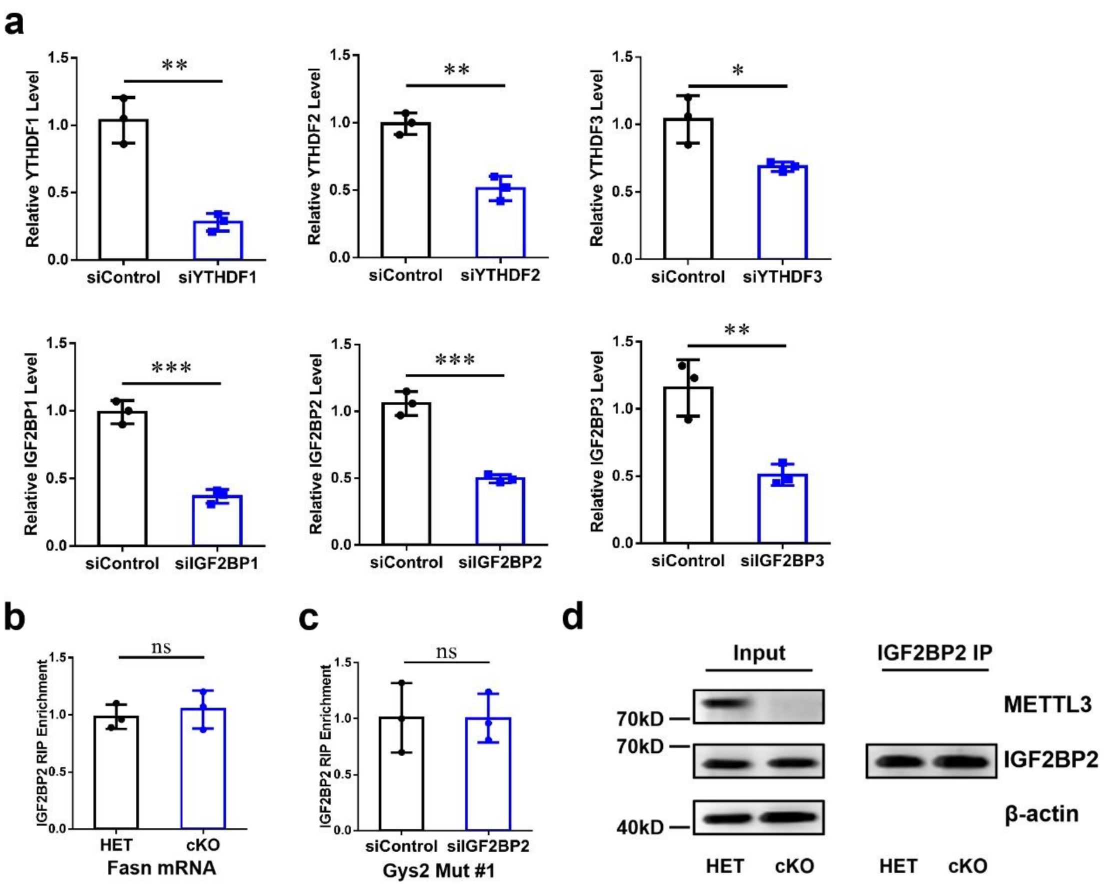

supplementary Figure 4. related to Figure 4. N6-methyladenosine stabilizes *Gys2* mRNA in an IGF2BP2 dependent manner. (a) qRT-PCR assay of candidate m6A readers' relative mRNA level (β-actin as reference) in siControl and indicated siRNAs. n=3 independent experiments. Data are presented as mean values +/- SEM. Two-sided Student's t test was performed to determine a difference among groups. *, p = 0.028 in YTHDF3; **, p = 0.002 in YTHDF1, p = 0.002 in YTHDF2, p = 0.007 in IGF2BP3; ***, p < 0.001 in IGF2BP1 and IGF2BP2. (b) enrichment of *Fasn* mRNA in mettl3-HET or cKO hepatocytes by IGF2BP2-RIP-qPCR. Two-sided Student's t test was performed to determine a difference between columns. ns, not significant. n=3 animals. Data are presented as mean values +/- SEM. (c) enrichment of Flag-Gys2-CDS Mut #1 in siControl or siIGF2BP2 Hepa1-6 cells by IGF2BP2-RIP-qPCR. n=3 animals. Data are presented as mean values +/- SEM. Two-sided Student's t test was performed to determine a difference among groups. ns, not significant. (d) Western blotting assay of indicated genes' protein levels in IGF2BP2-RIP. This experiment was repeated independently with similar results at least 3 times. Source data are provided as a Source Data file.

supplementary Table 1. Primers used for genotyping analysis.

Target	Forward Primer	Reverse Primer
Mettl3 loxP	AAAAGGCAATGTGCTTCTATGCCCG	TGCACGATGATAAAAGCCACTGTAAC
Albumin-Cre	TGGCAAACATACGCAAGGG	CGGCAAACGGACAGAAGCA

supplementary Table 2. Primers used for real-time PCR analysis.

Target	Forward Primer	Reverse Primer
mature Gys2 (mouse)	ACCAAGGCCAAAACGACAG	GGGCTCACATTGTTCTACTTGA
Mettl3 (mouse)	CTGGGCACTTGGATTTAAGGAA	TGAGAGGTGGTGTAGCAACTT
Aqp8 (mouse)	TGTGTAGTATGGACCTACCTGAG	ACCGATAGACATCCGATGAAGAT
Ythdf1 (mouse)	ACAGTTACCCCTCGATGAGTG	GGTAGTGAGATACGGGATGGGA
Ythdf2 (mouse)	GAGCAGAGACCAAAAGGTCAAG	CTGTGGGCTCAAGTAAGGTTC
Ythdf3 (mouse)	CATAGGGCAACAGAGGAAACAG	ATCTCCAGCCGTGGACCAT
Igf2bp1 (mouse)	CGGCAACCTCAACGAGAGT	GTAGCCGGATTTGACCAAGAA
Igf2bp2 (mouse)	GTCCTACTCAAGTCCGGCTAC	CATATTCAGCCAACAGCCCAT
Igf2bp3 (mouse)	CCTGGTGAAGACGGGCTAC	TCAACTTCCATCGGTTTCCCA
Fasn (mouse)	GGAGGTGGTGATAGCCGGTAT	TGGGTAATCCATAGAGCCCAG
β-actin (mouse)	GAGACCTTCAACACCCCAGC	ATGTCACGCACGATTTCCC
exogenous Gys2 (Flag-tag)	TTACAAGGATGACGACGAT	GGTCACCTCCCAAGAAAC
GFP	AGCAAAGACCCCAACGAGAA	TCGTCCATGCCGAGAGTGAT
pre-Gys2 (mouse)	CCCTGTGGAAGACTTACTG	GACACCTTCAGAGCCAAT
18s (mouse)	CCCGAAGCGTTTACTTTGA	ACTTTGGTTTCCCGGAAG
Mettl3 (rat)	CTGGGCACTTGGACTTAAGGAA	TGAGAGGTGGTGTAGCAACTT
Gys2 (rat)	CGCACGTGTTTACCACAGTGT	CAGGCTTCCTCTTCAGCATGT

β -actin (rat)	CCCATCTATGAGGGTTACGC	TTTAATGTCACGCACGATTTC

supplementary Table 3. siRNA used in this study.

Target	Sense (5' to 3')
siControl	UUCUCCGAACGUGUCACGUTT
YTHDF1 (mouse)	CCCGUAUCUCACUACCUAUTT
YTHDF2 (mouse)	GGGAUUGACUUCUCAGCAUTT
YTHDF3 (mouse)	GCAGUGGUAUGACUAGCAUTT
IGF2BP1 (mouse)	GCCAGCACAUCAAACAACUTT
IGF2BP2 (mouse)	GCCGCAUGAUUCUUGAGAUTT
IGF2BP3 (mouse)	GGCAGAGGAUUCGUAAACUTT
