# Supplementary material for: N6-methyladenosine modification governs liver glycogenesis by stabilizing the glycogen synthase 2 mRNA
Source: Nat Commun. 2022 Nov 17;13:7038. doi: 10.1038/s41467-022-34808-2 (PMC9671881; doi:10.1038/s41467-022-34808-2)
Supplement: Supplementary file 3 — Reporting Summary [file 41467_2022_34808_MOESM3_ESM.pdf]

nature portfolio

Corresponding author(s): Rui Zhang

Last updated by author(s): Nov 1, 2022

Reporting Summary

Nature Portfolio wishes to improve the reproducibility of the work that we publish. This form provides structure for consistency and transparency in reporting. For further information on Nature Portfolio policies, see our Editorial Policies and the Editorial Policy Checklist.

Statistics

For all statistical analyses, confirm that the following items are present in the figure legend, table legend, main text, or Methods section.

n/a	Confirmed	
☐	☒	The exact sample size (*n*) for each experimental group/condition, given as a discrete number and unit of measurement
☐	☒	A statement on whether measurements were taken from distinct samples or whether the same sample was measured repeatedly
☐	☒	The statistical test(s) used AND whether they are one- or two-sided<br>*Only common tests should be described solely by name; describe more complex techniques in the Methods section.*
☒	☐	A description of all covariates tested
☐	☒	A description of any assumptions or corrections, such as tests of normality and adjustment for multiple comparisons
☐	☒	A full description of the statistical parameters including central tendency (e.g. means) or other basic estimates (e.g. regression coefficient) AND variation (e.g. standard deviation) or associated estimates of uncertainty (e.g. confidence intervals)
☐	☒	For null hypothesis testing, the test statistic (e.g. *F*, *t*, *r*) with confidence intervals, effect sizes, degrees of freedom and *P* value noted<br>*Give P values as exact values whenever suitable.*
☒	☐	For Bayesian analysis, information on the choice of priors and Markov chain Monte Carlo settings
☒	☐	For hierarchical and complex designs, identification of the appropriate level for tests and full reporting of outcomes
☒	☐	Estimates of effect sizes (e.g. Cohen's *d*, Pearson's *r*), indicating how they were calculated

Our web collection on statistics for biologists contains articles on many of the points above.

Software and code

Policy information about availability of computer code

Data collection	The amplification signal of qPCR data was acquired by Bio-Rad CFX Manager 3.1. MeRIP-Seq was performed by Cloudseq Biotech Inc. (Shanghai, China) according to the published procedure (Meyer et al., 2012) with slight modifications. The library quality was evaluated with BioAnalyzer 2100 system (Agilent Technologies, Inc., USA). Library sequencing was performed on an illumina Hiseq instrument with 150bp paired-end reads. Paired-end reads were harvested from Illumina HiSeq 4000 sequencer, and were quality controlled by Q30. After 3' adaptor-trimming and low-quality reads removing by cutadapt software (v1.9.3). First, clean reads of all libraries were aligned to the reference genome (UCSC MM10) by Hisat2 software (v2.0.4). Methylated sites on RNAs (peaks) were identified by MACS software (Version 1.4.2). Differentially methylated sites were identified by diffReps (Version 1.55.6). These peaks identified by both softwares overlapping with exons of mRNA were figured out and chosen by home-made scripts. GO and Pathway enrichment analysis were performed by the differentially methylated protein coding genes.<br>Figure 3a-b: Motif analysis was performed using HOMER (Version 4.10) to search motifs in each set of m6A peaks. Metagene profiles were generated as described. Peak density plot was visualized by R package Trumpet (https://github.com/skyhorsetomoon/Trumpet).
Data analysis	Microsoft excel 2016 and GraphPad Prism (Version 6.02) were used to calculate mean, standard error and P value. Staining intensities were measured using Image J software (Version 1.50i; National Institutes of Health, Bethesda, MD, USA)

For manuscripts utilizing custom algorithms or software that are central to the research but not yet described in published literature, software must be made available to editors and reviewers. We strongly encourage code deposition in a community repository (e.g. GitHub). See the Nature Portfolio guidelines for submitting code & software for further information.

Data

Policy information about availability of data

All manuscripts must include a data availability statement. This statement should provide the following information, where applicable:

- Accession codes, unique identifiers, or web links for publicly available datasets
- A description of any restrictions on data availability
- For clinical datasets or third party data, please ensure that the statement adheres to our policy

Source data are provided with this paper. Source data for Figures 1a, 1c, 1d, 2a, 2c, 2d, 2f, 2g, 3e, 3f, 3h, 4a, 4b, 4c, 4e, 4g, 4h, 5b, 5c, 5d, 5f, 5g, 5h, 6a, 6b, 6c, 6d, 6e, 6f and Supplementary Figures 1b, 1c, 1e, 2b, 2c, 2e, 3d, 3e, 3f, 3g, 3h, 4a, 4b, 4c are provided as Source Data file. Unprocessed original scans of blots (Figures 2b, 3g, 4f, 5a and Supplementary Figures 1d, 4d) are also shown in Source Data file. RNA-sequencing and m6A-RIP sequencing raw data and processed expression matrix are uploaded to GEO DataSets under accession code GSE207566 (https://www.ncbi.nlm.nih.gov/geo/query/acc.cgi?acc=GSE207566). The sequencing reads were mapped to themousemm10 genome. A different-age mouse liver RNA-seq dataset (GSE58827) was download from GEO datasets (https://www.ncbi.nlm.nih.gov/geo/query/acc.cgi?acc=GSE58827). All other data analyzed or generated in this study are provided along with the article.

Human research participants

Policy information about studies involving human research participants and Sex and Gender in Research.

Reporting on sex and gender	N/A
Population characteristics	N/A
Recruitment	N/A
Ethics oversight	N/A

Note that full information on the approval of the study protocol must also be provided in the manuscript.

Field-specific reporting

Please select the one below that is the best fit for your research. If you are not sure, read the appropriate sections before making your selection.

☒ Life sciences ☐ Behavioural & social sciences ☐ Ecological, evolutionary & environmental sciences

For a reference copy of the document with all sections, see nature.com/documents/nr-reporting-summary-flat.pdf

Life sciences study design

All studies must disclose on these points even when the disclosure is negative.

Sample size	The sample size for each experiment was described in Figure Legends. Generally speaking, sample sizes were determined by similar literatures and in respect with the ethical criteria to use the minimal number of each experiment. Sample sizes in mouse and rat experiments were determined to get biological meaningful results, and thus enough to reflect the huge differences between Control and Mettl3-cKO mice. Detailed mice and rats numbers in each experiment were determined by the number of mice or rats born in the cages designed for indicated experiments. Sample sizes for qPCR, western blot, dual-luciferase report assay, and other cell and molecular experiments were designed for at least three samples in each group for analysis.
Data exclusions	No data were excluded from analysis.
Replication	Experiments were independently at least three independent biological or experimental replicates . All attempt to reproduce the results were successful.
Randomization	Mice with different genotypes were randomly divided into different groups. For example, when Control or cKO mice were divided into two groups, each individual was chosen just randomly and allocated to different groups. Likewise, for experiments other than those involving mice, samples were also randomly allocated into different groups.
Blinding	H&E staining, PAS staining, IHC staining and mouse/rat experiments in this article were performed and analyzed by investigators who were blinded for experimental designs. Blinding was not performed for cell-related experiments because the investigator must know the treatment for each group. For western blot, qPCR, MeRIP-qPCR, genotyping, and dual-luciferase report assay experiments, the investigator was not blinded since these experiments were conducted by the same person.

Reporting for specific materials, systems and methods

We require information from authors about some types of materials, experimental systems and methods used in many studies. Here, indicate whether each material, system or method listed is relevant to your study. If you are not sure if a list item applies to your research, read the appropriate section before selecting a response.

Materials & experimental systems

n/a	Involved in the study
☐	☒ Antibodies
☐	☒ Eukaryotic cell lines
☒	☐ Palaeontology and archaeology
☐	☒ Animals and other organisms
☒	☐ Clinical data
☒	☐ Dual use research of concern

Methods

n/a	Involved in the study
☒	☐ ChIP-seq
☒	☐ Flow cytometry
☒	☐ MRI-based neuroimaging

Antibodies

Antibodies used

Antibodies used in western blotting:
anti-METTL3 (1:1000, ab195352, 18810, abcam);
anti-GYS2 (1:2000, 22371-1-AP, proteintech);
anti-IGF2BP2 (1:1000, 11601-1-AP, proteintech);
anti-GFP (1:1000, 50430-2-AP, proteintech);
anti-FLAG tag (1:1000, F1804, Sigma-Aldrich);
anti-β-actin (1:4000, SAB1305554, Sigma-Aldrich);
HRP-conjugated anti-mouse IgG (1:10000, 7076, Cell Signaling Technology);
HRP-conjugated anti-rabbit IgG (1:10000, 7074, Cell Signaling Technology).

Antibodies used in MeRIP-qPCR:
anti-N6-methyladenosine (m6A), clone 17-3-4-1, from Millipore kit (17-10499), 5 μg per sample;
Normal Mouse IgG, from Millipore kit (17-10499), 5 μg per sample;
anti-IGF2BP2, 11601-1-AP, proteintech, 5 μg per sample;
Rabbit IgG, 30000-0-AP, proteintech, 5 μg per sample.

Antibodies used in IHC:
anti-GYS2 (1:100, 22371-1-AP, proteintech).

Validation

All of these antibodies are commercially available and have been test to recognize for both human and mouse species. The quality control and validation are provided by the manufacturer's websites:
Antibodies used in western blotting:
1. anti-METTL3 (1:1000, ab195352, abcam); (https://www.abcam.com/mettl3-antibody-epr18810-ab195352.html).
2. anti-GYS2 (1:2000, 22371-1-AP, proteintech); (https://www.ptgcn.com/products/GYS2-Antibody-22371-1-AP.htm).
3. anti-IGF2BP2 (1:1000, 11601-1-AP, proteintech); (https://www.ptgcn.com/products/IGF2BP2-Antibody-11601-1-AP.htm).
4. anti-GFP (1:1000, 50430-2-AP, proteintech); (https://www.ptgcn.com/products/eGFP-Antibody-50430-2-AP.htm)
5. anti-FLAG tag (1:1000, F1804, Sigma-Aldrich); (https://www.sigmaaldrich.cn/CN/zh/product/sigma/f1804).
6. anti-β-actin (1:4000, SAB1305554, Sigma-Aldrich); (https://www.sigmaaldrich.cn/CN/zh/product/sigma/sab1305554).
7. HRP-conjugated anti-mouse IgG (1:10000, 7076, Cell Signaling Technology); (https://www.cellsignal.cn/products/secondary-antibodies/anti-mouse-igg-hrp-linked-antibody/7076).
8. HRP-conjugated anti-rabbit IgG (1:10000, 7074, Cell Signaling Technology); (https://www.cellsignal.cn/products/secondary-antibodies/anti-mouse-igg-hrp-linked-antibody/7074).

Antibodies used in MeRIP-qPCR:
1. anti-N6-methyladenosine (m6A), clone 17-3-4-1, from Millipore kit (17-10499); (https://www.emdmillipore.com/US/en/product/Magna-MeRIP-m6A-Kit-Transcriptome-wide-Profiling-of-N6-Methyladenosine,MM_NF-17-10499).
2. Normal Mouse IgG, from Millipore kit (17-10499); (https://www.emdmillipore.com/US/en/product/Magna-MeRIP-m6A-Kit-Transcriptome-wide-Profiling-of-N6-Methyladenosine,MM_NF-17-10499).
3. anti-IGF2BP2, 11601-1-AP, proteintech; (https://www.ptgcn.com/products/IGF2BP2-Antibody-11601-1-AP.htm).
4. Rabbit IgG, 30000-0-AP, proteintech; (https://www.ptgcn.com/products/IgG-control-Antibody-30000-0-AP.htm).

Antibodies used in IHC:
anti-GYS2 (1:100, 22371-1-AP, proteintech); (https://www.ptgcn.com/products/GYS2-Antibody-22371-1-AP.htm).

Eukaryotic cell lines

Policy information about cell lines and Sex and Gender in Research

Cell line source(s)

Hepa 1-6 and HEK-293T cells were purchased from the American Type Culture Collection (ATCC).

Authentication	Cell lines were not authenticated by ourselves.
Mycoplasma contamination	All cell lines were tested to be mycoplasma negative.
Commonly misidentified lines (See ICLAC register)	No cell line listed in ICLAC was used.

Animals and other research organisms

Policy information about studies involving animals; ARRIVE guidelines recommended for reporting animal research, and Sex and Gender in Research

Laboratory animals	Methyltransferase-like 3 (Mettl3)-floxed, Albumin (Alb)-Cre, Alb-cre Mettl3-/- (wild-type, WT), Alb-cre Mettl3fl/- (HET) and Alb-cre Mettl3fl/fl (KO) mice were used. The wild-type Sprague-Dawley (SD) rats were used. All mice and rats were used at 4–10 weeks of age. Mice and rats were housed with a light cycle of 12h/12h at 23℃± 2℃ and 50%±10% humidity. All the mice and rats were maintained under specific pathogen-free conditions and used in accordance with the animal experimental guidelines set by the Institute of Animal Care and Use Committee.
Wild animals	The study did not involve wild animals.
Reporting on sex	Unless otherwise noted, male mice and rats were used in this study.
Field-collected samples	The study did not involve field-collected samples.
Ethics oversight	All animal procedures used in this experiment were performed in accordance with protocols approved by the Animal Experiment Administration Committee of Fourth Military Medical University.

Note that full information on the approval of the study protocol must also be provided in the manuscript.
